# And the Doctor Said

**DOI:** 10.1007/s10912-025-09991-0

**Published:** 2025-11-07

**Authors:** Makeen Yasar

**Affiliations:** College of Medicine, Charles R. Drew University of Medicine and Science, Los Angeles, United States

*“Don’t you know that anybody from your side of the tracks dies quicker anyways?”*

& I didn’t realize how prescriptive death sentences were these days wrapped in the curves of some numbered zip code// but I have heard of doctors writing prescriptions for death to the sound of the Grim Reaper’s sharpening scythe// & did you know that Black people are more likely to die from heart disease than any other race// & they tried to say it was only cause of the slave scraps we ate called soul food// & they tried to say it was because we held more salt in our bodies than any other race// & how wrong all that was// & maybe, just maybe// it was a Freudian slip from the guilt trip of remembering all the dead Africans they drowned in the salt of the Atlantic// & the stress is passed down by the generation// & our genes hold the trauma over generations// & I couldn’t throw a rock far enough to hit a doctor’s office for miles in the neighborhood David grew up in

& there has never been a Black woman whose life I did not fear for// & the same for any baby she did not yet have// & the irony is that Shelly only lost Carson after he was born// & did you know that half of all families investigated by CPS are Black// & when Ameer died// the rumor has it the doctors dragged his remains underneath the bed to Frankenstein him into a new boogeyman// to scare all the kids in Children’s Hospital whose parents did not have insurance

& did you know that insurers and business people with no medical degrees// sit on councils to decide who does and does not receive care based on a card with a policy number// & that hospitals can stand over someone dying // slowly // charging them thousands // & pass their corpse to the charge nurse// to send them home in a body bag with their belongings along with their discharge papers// & I’m not saying all doctors are bad// but damnit you’re a bad one if you don’t wanna blow this **** up

because how cruel it is none of us is guaranteed anything that might heal us in a land birthed out of our death// legacy of greed unraveling the oath to do no harm// & that doctors only become salves on the wounds of our people by grinding their egos into crushed herbs of repentance

& in spite of all this, I tell my grandmother I am becoming a doctor// & her diseases cower away// & my father never found much comfort in the arms of most things white// let alone a white coat// but how proud of me he was for showing him how a stethoscope on my neck wasn’t a noose in disguise// & this after him being just an arms length away from a bullet shot by some St. Louis rednecks// who thought it fun to make target practice out of a group of Black boys playing basketball// & I say that to say// that these things// are only two sides// of the same coin// depending on the day

## Data Availability

No datasets were generated or analysed during the current study.

